# Genetic polymorphisms of matrix metalloproteinases 1–3 and their inhibitor are not associated with premature labor

**DOI:** 10.4155/fsoa-2018-0047

**Published:** 2018-08-24

**Authors:** Konstantinos Lathouras, Srdjan Saso, Menelaos Tzafetas, Kallirroi Kalinderi, Styliani Fidani, Vassiliki Zournatzi, Maria Kyrgiou, Christina Fotopoulou, Sadaf Ghaem-Maghami, Ioannis Tzafetas

**Affiliations:** 1 Second Department of Obstetrics & Gynaecology, Aristotle University of Thessaloniki, Greece; 2 Imperial College Healthcare NHS Trust, London, UK; 3 Imperial College London, London, UK; 4 Department of General Biology, Aristotle University of Thessaloniki, Greece

**Keywords:** genetic polymorphism, matrix metalloproteinases, preterm labor

## Abstract

**Aim:**

Extracellular matrix metalloproteinases (MMPs) and their inhibitors (tissue inhibitors of metalloproteinases [TIMPs]) are involved in the breakdown of fetal membranes before delivery. Our aim was to investigate the occurrence of any polymorphism on genes coding for *MMPs 1-3* and *TIMP 2* in preterm laboring patients as a potential source of this phenomenon. This question has not been studied before.

**Methodology & results:**

A prospective population study was performed in a Greek university hospital. Group A (control) included 66 women with no symptoms of premature labor. Group B (research) comprised 66 women, exhibiting signs of threatened preterm labor. No statistically significant difference in polymorphism, both in the distribution of genotype as well as allele frequencies, was detected between the two groups. This also applied to gestational age less or greater than 32 weeks.

**Conclusion:**

Gene polymorphisms of *MMP 1–3* and *TIMP 2* are not associated with premature rupture of membranes/contractions, as well as gestational age at preterm labor.

## Lay abstract

Matrix metalloproteinases (MMPs) and their blockers (tissue inhibitors of metalloproteinases [TIMPs]) are two types of substances involved in the breakdown of the envelope that protects the baby before delivery. Our aim was to investigate any variation in the genes of these substances in patients who go into labor before 37 weeks. The study was performed in the outpatient antenatal and fetomaternal clinics in a Greek University Hospital. The gene polymorphisms of *MMP 1-3* and *TIMP 2* are not associated with premature rupture of membranes or contractions, as well as gestational age at preterm labor. More studies are required to further investigate other possible polymorphisms.

Labor occurring before 37th week of gestation is characterized as premature or preterm [1]. It may be initiated by two phenomena: rupture of fetal membranes, and commencement of uterine contractions. When rupture of membranes occurs prior to 37th week of gestation, it is called ‘preterm prelabour rupture of membranes’ (PPROM). It can indicate threatened preterm labor, which may in turn endanger the pregnancy outcome. Premature birth places the fetus at great risk. Besides their significantly lower birth weight compared with full-term newborns, more than 80% of premature newborns had to be admitted to the Neonatal Intensive Care Unit. Furthermore, it was observed that more than half of the women who delivered prematurely had to do so by a cesarean section, instead of experiencing a natural delivery. This leads to an increased risk of future cesarean sections, with added morbidity and potential mortality, as well as financial costs for the health service.

All these reasons clearly demonstrate the significance of discovering the involvement of some disorder or pathology on a biochemical and genetic level related to threatened premature labor. The strength, elasticity and integrity of fetal membranes play an important role in preventing PPROM. These three properties are influenced by various proteins of the extracellular matrix (ECM), such as metalloproteinases as well as their inhibitors. Their effect can either be favorable, as exemplified by the breakdown of fetal membranes prior to a physiological term delivery, or precarious as when this breakdown occurs prematurely (PPROM) [2,3].

Matrix metalloproteinases (MMPs) are proteolytic enzymes, specifically endopeptidases. They are members of the broader protease metzincin super-family. They are involved in a number of physiological (implantation of the fertilized ovum, embryogenesis, morphogenesis, reproduction, lysis and tissue remodeling, ovulation, labour and rupture of fetal membranes) and pathological processes (wound healing, arthritis, multiple sclerosis, cirrhosis, atherosclerosis, heart failure, myocardial infarction, cancer and metastasis) [4–13]. MMPs are inhibited primarily by two inhibitors: macroglobulin α2; and tissue inhibitors of MMPs (TIMPs). Production of both MMPs and TIMPs occurs in various cells and tissues. Expression of metalloproteinases, for instance, can take place in cells of mesenchymal origin such as fibroblasts or smooth muscle cells, but also in macrophages, keratinocytes, endothelial cells, osteoblasts, neutrophils and various tumor cells [14,15].

MMPs mainly target parturition. Both term and preterm parturition require extensive ECM remodeling where MMPs play a crucial role in the breakdown of ECM proteins. Examples of target tissues for ECM remodeling include ripening and dilation of the cervix, rupture of fetal membranes and detachment of the placenta from the maternal uterus.

MMPs and TIMPs are encoded by genes. Genetic polymorphism refers to the diversity of genes as alleles at 1% or more of the population. The significance of a polymorphism is that it generates genetic variation among organisms. This is achieved by creating different end products of genes, which alter, for instance, enzymatic activities and ligand affinity, or in some cases, even by producing a new phenotype. Diagnosing women with a type of polymorphism in one of the genes which codes for MMPs or TIMPs, in other words, an abnormality in their genotypes or alleleic frequencies, would help detect pregnancies at increased risk for premature labor. This would be both prenatally or during an early stage of gestation. The possibility of early screening for such abnormalities could be later developed and used as a precaution. Women with such genetic polymorphisms could be placed among a high-risk population, resulting in closer and more frequent prophylactic monitoring. This would simultaneously ensure a safer course of the pregnancy for both the mother and the fetus. Furthermore, attempts for therapeutic intervention could be made in the future, as genetic manipulation is nowadays a rapidly developing field.

## Study objective

The main study objective was to examine whether pregnant women with symptoms of preterm labor contain polymorphic genes encoding for MMPs and TIMPs. We hypothesize that locating the aforementioned polymorphisms may lead to a better understanding of preterm labor.

## Materials & methods

### Patient groups

This was a prospective, cross-sectional population study. The study population was divided into two groups. Group A (control group) comprised of 66 pregnant women at 24th–37th week of gestation and without any symptoms of premature labor. To ensure a healthy state in the antenatal period, blood samples were taken during gestation weeks 11–14 and 24–37. Group B (research group) comprised of 66 pregnant women at 24th–37th week of gestation, exhibiting the classic symptoms of threatened preterm labor (preterm uterine contractions and/or premature spontaneous rupture of fetal membranes). Furthermore, Group B was divided into two further groups defined by a gestational age less or more than 32 weeks.

The patients of the control group were selected from the out-patient antenatal clinic of the Department of Obstetrics and Gynaecology, Hippokratio Hospital, Aristotle University, Thessaloniki, Greece. Research group patients were recruited from the feto–maternal unit of the same hospital.

### Inclusion & exclusion criteria

Inclusion criteria of the control group were pregnant women from 24 to 37 weeks of gestation without any symptoms of preterm labor, previous history of preterm labor, multiple pregnancies that delivered after 37 weeks of gestation and no other significant medical history. Inclusion criteria for the research group were preterm deliveries below 37 weeks of gestation, and unprovoked onset of threatened preterm labor, either by preterm, prelabor rupture of amniotic membranes, onset of uterine contractions or both.

Exclusion criteria for the research group were pregnant women with any obvious risk factors that could contribute to a preterm delivery. Examples are previous history of preterm birth, multiple pregnancy, cervical length <25 mm, chorioamnionitis, infection of the lower genital tract, infections of the upper and lower urinary tract, significant pregnancy pathology or severe systemic disease, anatomical uterine abnormalities, positive vaginal or urinary microbiology cultures. Women with previous history of preterm birth were excluded as we wanted to minimize the risk of bias. Such women have an increased risk of future preterm birth and would therefore influence the results.

### Sample collection

A detailed medical history was obtained from all participants, including a physical examination. The following variables were recorded: fundal height, auscultation of heart sounds, arterial pressure, pulse rate, body temperature and somatometric data. Pathology profiling was split into five groups: full blood count (hematocrit, hemoglobin, platelets, leukocyte and erythrocyte count), blood type including rhesus status, biochemistry (TSH, FT_3_, FT_4_, glucose, urea, uric acid, creatinine, SGOT, SGPT, potassium, sodium, γGT, LDH and CRP), clotting (PT, PTT, INR, fibrinogen and D-dimer), and microbiology (urine culture and high vaginal swab). Further imaging was performed including: fetal growth, fetal biophysical profile and cervical length.

### Laboratory methodology

The genes for *MMPS 1–3* and *TIMP 2* were detected by performing a polymerase chain reaction (PCR). The restriction enzymes related to the study were Tth111I, Tsp45I, KpnI and AluI. DNA was isolated from the peripheral blood of the study participants. PCR was performed to amplify a specific DNA sequence in laboratory conditions. Following this, the samples underwent gel electrophoresis. Restriction Fragment Length Polymorphism (RFLP) was applied during which PCR products were digested using restriction enzymes. Subsequently, genotypes for each polymorphism were recorded for all patients of both groups. The polymorphisms that were studied and the above methodology are further elaborated upon in the Supplementary Material.

### Ethics review & statistics

The procedures that formed this study were assessed by the institutional review committee (Bioethics Committee, Medical School of Aristotle University, Thessaloniki, Greece; Number of Approval 5, Date: 06/07/2016). In addition, patients’ informed consent was obtained.

The study does not violate the policies and/or procedures established by journals such as those described in ‘Specific Inappropriate Acts in Publication Process’ and the protocol conforms to the provisions of the Declaration of Helsinki (as revised in Tokyo 2004), available at www.wma.net/en/30publications/10policies/b3/index.html

Quantitative variables are expressed as mean values (in addition to standard deviation, SD) or as a mean interquartile range (IQR). Qualitative variables are communicated as absolute and relative frequencies. For comparison, the Pearson's χ^2^ test and Fisher's exact test were used. Student *t*-test was used to compare mean values when distribution was normal and the Mann–Whitney test when distribution was abnormal. All p-values are ‘two-tailed’. Statistical significance was set at 0.05 and analysis was performed utilizing SPSS statistical software (version 19.0; IBM, London, UK).

## Results

### Patient characteristics

The sample consisted of 132 women (66 in Group A, i.e., control group and 66 in Group B, i.e., research group). The demographic and perinatal data, as well as the characteristics of the two groups are presented in Table 1. The mean age was 28.8 years (SD = 3.7 years) for Group A and 28.5 years (SD = 5.0 years) for Group B (p = 0.721). The two populations demonstrated no statistical significance with regards to parity, smoking, gynecological, or family history or episodes of hemorrhage during pregnancy. Regarding the symptomatology of premature labor, 50% of the study group had premature uterine contractions, 37.9% experienced PPROM and 12.1% presented with both symptoms.

**Table 1.** **Demographic, maternal and perinatal data and characteristics of Group A (control) and Group B (study).**

**Patient characteristics and outcomes**	**Group**		**p-value**

	**A n (%)**	**B n (%)**	
Age, mean (SD)	28.8 (3.7)	28.5 (5.0)	0.721^†^

Gestational week (samples were taken), mean (SD)	30 (4.0)	30 (3.8)	0.946^†^

Parity			

1	39 (59.1)	44 (66.7)	0.368^¶^

2–3	27 (40.9)	22 (33.3)	

Smoking			

No	61 (92.4)	58 (87.9)	0.381^¶^

Yes	5 (7.6)	8 (12.1)	

Positive gynaecological history			

No	56 (84.8)	61 (92.4)	0.170^¶^

Yes	10 (15.2)	5 (7.6)	

Positive family history			

No	63 (95.5)	66 (100.0)	0.244^§^

Yes	3 (4.5)	0 (0.0)	

Bleeding during pregnancy			

No	66 (100.0)	61 (92.4)	0.058^§^

Yes	0 (0.0)	5 (7.6)	

Abdominal or lumbar pain or sensation of suprapubic pressure			

No	56 (84.8)	35 (53.0)	<0.001^¶^

Yes	10 (15.2)	31 (47.0)	

Chorioamnionitis			

No	66 (100.0)	66 (100.0)	–

Yes	0 (0.0)	0 (0.0)	

Cervical length, mean (SD)	33.1 (4.1)	31.1 (4.9)	0.009^†^

Gestational age at delivery, median (IQR)	39 (38 - 40)	30 (26–34)	<0.001^‡^

Birth weight (gr), mean (SD)	3515 (258.9)	1810 (651.3)	<0.001^†^

Type of delivery			

Vaginal delivery	47 (71.2)	31 (47.0)	0.005^¶^

Cesarian section	19 (28.8)	35 (53.0)	

Neonatal Intensive Care Unit			

No	63 (95.5)	13 (19.7)	<0.001^¶^

Yes	3 (4.5)	53 (80.3)	

^†^Student's *t*-test

^‡^Mann–Whitney test

^¶^Pearson's χ^2^ test

^§^Fisher's exact test

IQR: Interquartile range; SD: Standard deviation.

### Pregnancy outcomes

None of the participants were affected by chorioamnionitis during pregnancy and the cervical length was more than 2.5 cm in both groups (mean value of length of cervix – Group A: 3.3 cm and Group B: 3.1 cm). The median gestational age at delivery was 39 weeks (IQR: 38–40) for the control group and 30 weeks (IQR: 26–34) for the study group, of which 65.2% went into premature labor at 32 weeks or earlier. Delivery by cesarean section was more frequent in the preterm group (53% as opposed to 28.8%; p = 0.005). The average birth weight was significantly lower among the preterm labor group. The proportion of newborns admitted to the Neonatal Intensive Care Unit was 80.3 and 4.5% in the control group.

### MMP 1–3 & TIMP 2

Vital signs and biochemical markers are presented in Table 2 with no statistically significant difference between the two groups. Table 3 shows the genotype and allele frequencies *of MMP 1–3* and *TIMP 2* of both groups. No statistically significant differences of polymorphism, both in the distribution of genotype and the allele frequencies between the two groups were detected.

**Table 2.** **General and biochemical characteristics.**

**Patient vital signs and blood profile**	**Group**		**p-value**

	**A Mean (SD)**	**B Mean (SD)**	
Systolic blood pressure (mmHg)	117.4 (8.3)	118.3 (9.3)	0.529^†^

Diastolic blood pressure (mmHg)	68.2 (5.8)	67.8 (6.0)	0.724^†^

Heart rate/min	79.8 (5.4)	80 (5.8)	0.889^†^

Temperature (Celcius)	36.8 (0.2)	36.7 (0.2)	0.272^†^

Hemoglobin	12 (0.7)	12.1 (0.6)	0.728^†^

White blood cell count	12 (1.8)	12.3 (1.6)	0.243^†^

Platelets	278.8 (78.4)	255.8 (77.3)	0.091^†^

Urea (mg/dl)	27.5 (9.4)	27 (10.1)	0.741^†^

Creatinine (mg/dl)	1.1 (0.2)	1.2 (0.3)	0.333^†^

Glucose (mg/dl)	89.4 (12.4)	87.1 (12.3)	0.275^†^

Thyroid stimulating hormone (μIU/ml)	2.8 (1.3)	2.8 (1.1)	0.761^†^

CRP, n (%)			

<5	66 (100.0)	61 (92.4)	0.058^‡^

>5	0 (0.0)	5 (7.6)	

^†^Student's *t*-test;

^‡^Pearson's χ^2^ test.

**Table 3.** **Genotype and allele frequencies of MMP 1, MMP 2, MMP 3 and TIMP 2. **

**Genotype and allele frequencies**	**Group**		**p-value**

	**A n (%)**	**B n (%)**	
MMP1			

A/A	27 (40.9)	31 (47.0)	0.191^†^

G/A	34 (51.5)	25 (37.9)	

G/G	5 (7.6)	10 (15.2)	

MMP2			

A/A	2 (3.0)	6 (9.1)	0.272^‡^

G/A	22 (33.3)	24 (36.4)	

G/G	42 (63.6)	36 (54.5)	

MMP3			

5A/5A	11 (16.7)	18 (27.3)	0.331^†^

5A/6A	40 (60.6)	34 (51.5)	

6A/6A	15 (22.7)	14 (21.2)	

TIMP2			

G/G	1 (1.5)	3 (4.5)	0.609^‡^

T/G	21 (31.8)	23 (34.8)	

T/T	44 (66.7)	40 (60.6)	

MMP1			

G/A	34 (51.5)	25 (37.9)	0.151^†^

MMP2			

G/A	22 (33.3)	24 (36.4)	0.715^†^

MMP3			

5A/6A	40 (60.6)	34 (51.5)	0.293^†^

TIMP2			

T/G	21 (31.8)	23 (34.8)	0.712^†^

^†^Pearson's χ^2^ test;

^‡^Fisher's exact test.

Genotypes and allelic frequencies of *MMP 1–3* and *TIMP 2*, in relation to gestational age less or greater than 32 weeks for the premature labor group are presented in Table 4. No statistically significant difference was found. The mean gestational age at the point of preterm labor demonstrated no statistically significant relation to *MMP 1* (p = 0.887) or *MMP 2* polymorphisms (p = 0.586). Finally, the mean gestational age at the point of preterm labor did not differ significantly in relation to *MMP 3* (p = 0.724) or *TIMP 2* polymorphisms (p = 0.736).

**Table 4.** **Genotype and allele frequencies of MMP 1-3 and TIMP 2 in relation to gestational age during delivery (Group B - study group).**

**Genotypes and their allele frequencies**	**Gestational week at time of delivery**		**p-value ^†^**

	**≤32 n (%)**	**>32 n (%)**	
MMP1			

Other	26 (60.5)	15 (65.2)	0.705

G/A	17 (39.5)	8 (34.8)	

MMP2			

Other	27 (62.8)	15 (65.2)	0.845

G/A	16 (37.2)	8 (34.8)	

MMP3			

Other	19 (44.2)	13 (56.5)	0.339

5A/6A	24 (55.8)	10 (43.5)	

TIMP2			

Other	28 (65.1)	15 (65.2)	0.993

T/G	15 (34.9)	8 (34.8)	

^†^Pearson's χ^2^ test.

## Discussion

### Background

Preterm labor can lead to difficulties concerning not only the mother and family in general, but also for public health as a whole. Three percent of pregnancies are complicated by preterm prelabour rupture of membranes (PPROM). In addition, PPROM is deemed responsible for approximately one third of all preterm births. With PPROM, early delivery, a situation where prenatal and neonatal complications are common, can be foreseen. Hence, the healthcare professional caring for this group of patients has an opportunity to intervene in a manner that can improve perinatal outcome [16].

The role of MMPs and their inhibitors in relation to rupture of fetal membranes before physiological delivery has already been established. MMPs can be classified into six subgroups: collagenases, gelatinases, stormelysins, membrane-type, metalloelastases and ‘other type’ [17]. We chose one type of MMP from the first three aforementioned groups, in view of literature findings that point to those six subgroups being prominent in preterm labor. MMP 1 is produced by mesenchymal cells such as fibroblasts and macrophages and is responsible for breaking down collagen type I–III. Collagen type III is present in amnion and mainly responsible for its integrity and durability. MMP 2 is responsible for breaking down fibronectin, and collagen type IV and VII. It is well established how fibronectin is related with respect to rupture of membranes. MMP 3 is responsible for breaking down collagen type IV and fibronectin. Their increased concentration in this clinical setting proves their involvement in the loss of fetal membrane integrity [18–21]. It has been shown that increased concentrations of *MMPs 1, 2 and 9*, coupled with simultaneous decrease in the concentration of *TIMP 1*, can indeed be measured when fetal membranes breakdown prematurely [22].

### Our findings

Although research aiming to unmask a direct ‘cause and effect’ relationship is still ongoing, questions still remain regarding the reason behind the seemingly spontaneous increase in the concentrations of certain metalloproteinases in some women of a premature gestational age. When excluding factors such as infections, unhealthy lifestyle and congenital and anatomical anomalies (maternal and fetal), the effect of genetics and that of genetic polymorphism in particular should be considered.

This original study looked into the existence of any such polymorphism in the genes coding for *MMPs 1, 2 and 3* and *TIMP 2*, in pregnant women with symptoms of preterm labor and compared the results of their genetic screening with those pregnant women with similar demographical characteristics (age, general health, absence of health and other risk factors) but with full-term pregnancies.

The results of this investigation indicated no statistically significant difference in genetic polymorphism for genes coding for *MMPs 1* (p = 0.887); 2 (p = 0.586); *3* (p = 0.724) or *TIMP 2* (p = 0.736) between women who had a premature delivery and those of the control group who delivered at full-term. Moreover, no significant differences in genotypes and alleleic frequencies were detected among women of the study group who delivered before 32 weeks of gestation and those who delivered after (*MMP-1*; p = 0.887; *MMP-2*; p = 0.586; *MMP-3*; p = 0.724; *TIMP-2*; p = 0.736). Therefore, the initial hypothesis that genetic polymorphism of the particular genes might play a role in the occurrence of preterm labor has to be rejected. Nevertheless, this only proves that no polymorphism exists in the specific genes but does not absolutely exclude it as a causative factor for the occurrence of PPROM and/or premature delivery. Further research could target the investigation of polymorphisms in other members of the metalloproteinase family and their inhibitors or other enzymes participating in the breakdown of fetal membranes.

To the best of our knowledge, this is the first population-based study looking at the characterization of polymorphisms of genes encoding for *MMPs and TIMPs*. It was done prospectively, involving a control group and a research group. Three genes were assessed, as well as their inhibitor. Importantly, the study has potentially identified a need to further research polymorphisms of genes that may be responsible for premature labor, in particular premature rapture of membranes. A large multicenter study to evaluate these effects and how such information may play a role in preventing premature labor and managing it if it occurs would be the next logical step.

### Limitations

The limitations also need outlining. The study was not a randomized controlled trial and the numbers were not statistically powerful to draw a fool-proof conclusion. With respect to the findings, we did not reveal a positive correlation. Furthermore, this study needs to be also recognized in the context of the recent research published by Zhang* et al.* [23]. They performed a genome-wide association study in a discovery set of samples obtained from 43,568 women of European ancestry to determine possible genetic associations with preterm birth. The authors found that variants at the EBF1, EEFSEC, AGTR2, WNT4, ADCY5 and RAP2C loci were associated with gestational duration and variants at the EBF1, EEFSEC and AGTR2 loci with preterm birth [23]. We feel that despite the discrepancy in the study populations between our study and the NEJM study, it is still worth publishing our results. First, confirmation of existing knowledge is always welcome. Second, some of the data were self-reported, which means an inevitable disagreement with medical records. Third, the authors in Zhang *et al*. could not distinguish spontaneous births from induced births. Fourth, all their participants were of European ancestry, leading to uncertainty whether the same loci are involved in gestational age among women of alternative ancestries [23]. Finally, our study, albeit small, is a case–control study, and we therefore have a comparison group.

## Conclusion

The key point of the current study is that the studied gene polymorphisms of *MMP 1–3* and *TIMP-2* do not appear to be associated with premature rupture of membranes or/and onset of preterm contractions. The specific gene polymorphisms studied here do not seem to correlate with the gestational age of preterm labor. This is the first time that these specific MMPs have been studied for potential polymorphisms.

## Future perspective

Although the present study did not manage to prove a correlation between specific polymorphisms of the genes of MMPs and their inhibitor, and premature rupture of membranes, a need is apparent to further investigate other possible polymorphisms. The identification of a causative correlation between isomorphisms of these genes and threatened preterm labor will possibly enable us to detect women at increased risk. This will allow for closer follow-up and effective and punctual management, resulting in either prevention or optimal outcome of preterm labor. Although not an outcome for this study, quantifying MMPs may be of interest in future work.

**Summary points**

- Extracellular matrix metalloproteinases (MMPs) and their inhibitors (TIMPs) are involved in the breakdown of fetal membranes before delivery.
- Our aim was to investigate the occurrence of any polymorphism on genes coding for *MMPs 1–3* and *TIMP 2* in preterm laboring patients as a potential source of this phenomenon.
- This question has not been studied before.
- The genes for *MMPs 1–3* and *TIMP 2* were detected by performing a polymerase chain reaction (PCR). Restriction Fragment Length Polymorphism was applied to digest PCR products using restriction enzymes.
- No statistically significant difference in polymorphism (*MMP 1–3* and *TIMP 2*), both in the distribution of genotype as well as allele frequencies were detected between the two groups.
- Genotypes and allelic frequencies of *MMP 1–3* and *TIMP 2* in relation to gestational age less/greater than 32 weeks for the premature labor group also demonstrated no statistically significant difference (p = 0.724/p = 0.736, respectively).
- The gene polymorphisms of *MMP 1–3* and *TIMP 2* are not associated with premature rupture of membranes/contractions, as well as age of pregnancy at preterm labor.
