# Supplementary material for: Genetic polymorphisms of matrix metalloproteinases 1–3 and their inhibitor are not associated with premature labor
Source: Future Sci OA. 2018 Aug 24;4(9):FSO332. doi: 10.4155/fsoa-2018-0047 (PMC6222277; doi:10.4155/fsoa-2018-0047)
Supplement: Supplementary file 1 [file fsoa-04-332-s1.docx]

**Appendix A**

The polymorphisms studied are as follows:

**MMP 1 (519 A / G)**

PCR program:

Thermal denaturation at 94°C for 5 minutes.

Hybridization of the primers 30 cycles at 94°C for 30 seconds, 60°C - 0.4°C / cycle for 30 seconds, 72°C for 45 seconds, followed by Polymerization at 72 ° C for 10 minutes

Starters

Forward: CATGGTGCTATCGCAATAGGGT

Reverse: TGCTACAGGTTTCTCCACACAC

Restriction enzyme: KpnI

Agarose gel electrophoresis 3.5%

Digestion products

G / G = 200

G / A = 200 + 176 + 24

A / A = 176 + 24

**MMP 2 (1575 G / A)**

PCR program

Thermal denaturation at 94°C for 5 minutes.

Hybridization of the primers 30 cycles at 94°C for 30 seconds, 60°C - 0.4°C / cycle for 30 seconds, 72°C for 45 seconds, followed by Polymerization at 72°C for 10 minutes.

PRIMERS

Forward: ACTGACTCTGGAAAGTCAGAGCA

Reverse: GGCACAGGGTGAGGGGATGG

Restriction enzyme: Tsp45I

Agarose gel electrophoresis 2%

Digestion products

A / A = 269

G / A = 269 + 156 + 113

G / G = 156 + 113

**MMP 3 (1171 5A → 6A)**

PCR program

Thermal denaturation at 94°C for 5 minutes.

Hybridization of the primers 30 cycles at 94°C for 30 seconds, 60°C - 0.4°C / cycle for 30 seconds, 72°C for 45 seconds, followed by Polymerization at 72°C for 10 minutes

Starters

Forward: GGTTCTCCATTCCTTTGATGGGGGGAAAGA

Reverse: CTTCCTGGAATTCACATCACTGCCACCACT

Restriction enzyme: TthlllI

Agarose gel electrophoresis 4%

Digestion products

5A/5Α=97+32

5A/6A=129+97+32

6A/6Α=129

**TIMP 2 (rs55743137)**

PCR program

Thermal denaturation at 94°C for 5 minutes.

Hybridization of the primers 30 cycles at 94°C for 30 seconds, 60°C - 0.4°C / cycle for 30 seconds, 72°C for 45 seconds, followed by Polymerization at 72°C for 10 minutes.

LEFT PRIMER CCTTTGAACATCTGGAAAGACAA

RIGHT PRIMER TAACCCATGTATTTGCACTTCCT

Restriction enzyme: AluI

Agarose gel electrophoresis 2%

Digestion products

T / T = 160

G / G = 108 + 52

T / G = 160 + 108 + 52

**Table A.** Polymorphisms investigated in the study.

|  | **MMP1 -519 A/G** | **MMP2 -1575 G/A** |
| --- | --- | --- |
| ***Denaturation*** | 94^o^C for 5 minutes | 94^o^C for 5 minutes |
| ***Hybridisation of primers*** | 30 cycles at 94^o^C for 30 seconds  60^o^-0.4^o^C/cycle for 30 seconds  72^o^C for 45 seconds | 30 cycles at 94^o^C for 30 seconds  65^o^-0.4^o^C/ cycle for 30 seconds  72^o^C for 45 seconds |
| ***Polymerisation*** | 72^o^C for 10 minutes | 72^o^C for 10 minutes |
| **Starters Forward** | CATGGTGCTATCGCAATAGGGT | ACTGACTCTGGAAAGTCAGAGCA |
| **Starters Reverse** | TGCTACAGGTTTCTCCACACAC | GGCACAGGGTGAGGGGATGG |
| **Restriction enzyme** | KpnI | Tsp45I |
| **Gel Electrophoresis** | 3.5% | 2% |
| **Digestion products** | G/G=200  G/A=200+176+24  A/A=176+24 | A/A=269  G/A=269+156+113  G/G=156+113 |
|  | | |
|  | **MMP3 -1171 5A🡪6A** | **TIMP2 rs55743137** |
| ***Denaturation*** | 94^o^C for 5 minutes | 94^o^C for 5 minutes |
| ***Hybridisation of primers*** | 30 cycles at 94^o^C for 30 seconds  65^o^-0.4^o^C/ cycle for 30 seconds  72^o^C for 45 seconds | 30 cycles at 94^o^C for 30 seconds  65^o^-0.4^o^C/ cycle for 30 seconds  72^o^C for 45 seconds |
| ***Polymerisation*** | 72^o^C for 10 minutes | 72^o^C for 10 minutes |
| **Starters Forward** | GGTTCTCCATTCCTTTGATGG  GGGGAAAGA | CCTTTGAACATCTGGAAAGACAA |
| **Starters Reverse** | CTTCCTGGAATTCACATCACT  GCCACCACT | TAACCCATGTATTTGCACTTCCT |
| **Restriction enzyme** | TthlllI | AluI |
| **Gel Electrophoresis** | 4% | 2% |
| **Digestion products** | 5A/5Α=97+32  5A/6A=129+97+32  6A/6Α=129 | T/T=160  G/G=108+52  T/G=160+108+52 |
